# An Ultra-Performance Liquid Chromatography–Tandem Mass Spectrometry (UPLC–MS/MS) Method for Qualifying DAPB in Rat Plasma and Application to Pharmacokinetic Studies

**DOI:** 10.3390/molecules29020541

**Published:** 2024-01-22

**Authors:** Bei Qin, Yunmei Chen, Kuan Yang, Rong Wang, Lili Yu, Nana Wang, Shaojing Liu

**Affiliations:** 1 Xi’an Key Laboratory for Research and Development of Innovative Multi-Target Antihypertensive Drugs, Xi’an Innovative Antihypertensive Drugs International Science and Technology Cooperation Base, Xi’an Medical University, Xi’an 710021, China; yunmeichen@xiyi.edu.cn (Y.C.); yangkuan@xiyi.edu.cn (K.Y.); wangrong@xiyi.edu.cn (R.W.); yulili@xiyi.edu.cn (L.Y.); wangnana@xiyi.edu.cn (N.W.); liushaojing@xiyi.edu.cn (S.L.); 2 Institute of Drug Research, Xi’an Medical University, Xi’an 710021, China; 3 College of Pharmacy, Xi’an Medical University, Xi’an 710021, China

**Keywords:** UPLC–MS/MS, pharmacokinetic study

## Abstract

DAPB, a new molecule including danshensu, borneol, and a mother nucleus of ACEI (Angiotensin-converting enzyme inhibitors), is being developed as an antihypertensive candidate compound. A rapid, accurate, and sensitive ultra-performance liquid chromatography–tandem mass spectrometry (UPLC–MS/MS) method was established and validated for the determination of DAPB in rat plasma. Chromatographic separation was performed on an Agilent SB-C18 column after protein precipitation by acetonitrile with a mobile phase consisting of acetonitrile and deionized water with 0.02% formic acid and 5 mM NH_4_F (*v*/*v*) at a flow rate of 0.2 mL/min. Quantification was performed using electrospray positive ionization mass spectrometry in the multiple reaction monitoring (MRM) mode. The method was linear over the range of 2–1000 ng/mL. The intra- and inter-day precision was within 12%, with accuracies less than 7%. Stability was within the acceptable limits under various storage and processing conditions. No apparent matrix effect was detected. The validated method was applied to the pre-clinical pharmacokinetic study of DAPB after oral administration of 30 mg/kg and intravenous administration of 6 mg/kg in rats.

## 1. Introduction

Hypertension is a chronic disease characterized by continuous rising arterial blood pressure that has become a global public health issue [1]. As a disease with high mortality risk worldwide, hypertension not only causes a series of cardiovascular and cerebrovascular diseases but also leads to damage to the structure and function of important organs such as the heart, brain, and kidneys, posing a significant threat to the lives and health of people [2,3]. The aim of antihypertensive therapy is not only to improve the value of blood pressure but also to effectively prevent or delay the occurrence of hypertension-associated complications.

Angiotensin-converting enzyme inhibitors (ACEIs), suppressing the activity of angiotensin-converting enzyme (ACE) that is responsible for the conversion of angiotensin I to angiotensin II, have been the first-line drugs for improving hypertension [4,5]; however, ACEIs have side effects such as an irritating dry cough, angioedema, and abnormal renal function, which seriously affect the quality of life and health of patients [6,7,8]. Therefore, developing a new ACEI drug with fewer side effects is urgent for the treatment of hypertension [9].

**Figure 1:**
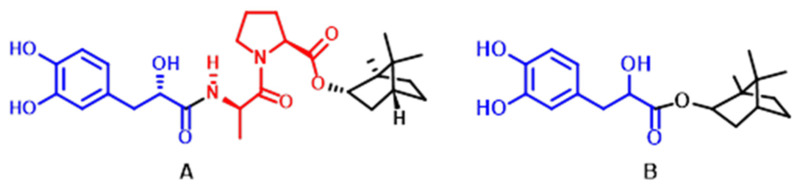
Chemical structures of DAPB (**A**) and DBZ (**B**).

DAPB, a novel candidate drug with antihypertensive activity named (2*R*)-3-(3,4-dihydroxyphenyl)-2-hydroxypropionyl-*L*-alanyl-*L*-proline bornyl ester, was synthesized from danshensu (marked with a blue line in Figure 1); borneol (marked with a black line in Figure 1); and proline (pro; marked with a red line in Figure 1), the active group of ACEI, which can bind to ACE resulting in the inactivation of ACE [10,11]. The structural representation of DAPB is illustrated in Figure 1 [12]. Borneol, an upper ushering drug that possesses the potential to promote the penetration of the drug through biological barriers to reach the brain, was connected to the C terminal of the Pro residue through an ester bond [13]. The purpose of choosing danshensu is that it not only has the effect of lowering blood pressure and protecting organs but also can coordinate with the zinc ions in the active center of the ACE enzyme [14]. The binding of danshensu and the alanine terminal of the Pro residue through an amide bond may achieve a synergistic hypotensive effect while also protecting organs such as blood vessels [15] and the heart [16,17], kidney [18], and liver [19,20]. It is reported that DAPB decreased the systolic blood pressure (SBP) and diastolic blood pressure (DBP) in SHR rats by 50 mmHg and 35 mmHg, respectively [12]. The mechanism by which DAPB improves hypertension may be related to repressed RAAS and elevated NO production. Moreover, DAPB could protect cardiac function by improving the morphology of cardiac cells and relieving the development of cardiac fibrosis in SHR rats, showing that DAPB possesses the function of organ protection [21].

Preclinical pharmacokinetics has a significant impact on the behavior and pharmacokinetic characteristics of potential candidates in the body, providing advice for accelerating further drug research and development [22]. As a new compound, the pharmaceutical analysis and pharmacokinetic study of DAPB have not yet been reported. The qualified bio-analytical method is essential for pharmacokinetic study, and the LC–MS/MS method has emerged as the preferred method for the quantitative analysis of biological samples [23]. A high-performance liquid chromatography (HPLC) method has been validated for the determination of DAPB in methanol, but it has failed in the analysis of DAPB in plasma samples due to its low sensitivity and significant matrix effect in UV detection [24]. To our knowledge, there is no validated analytical method for the determination of DAPB in complicated biological samples. The aim of this work was to develop and validate a sensitive, specific, and accurate method for quantifying DAPB in rat plasma. The validated method was applied in the pharmacokinetic study of analytes after intravenous injection (6 mg/kg) and oral administration (30 mg/kg) in rats.

## 2. Results

### 2.1. Method Development

**Figure 2:**
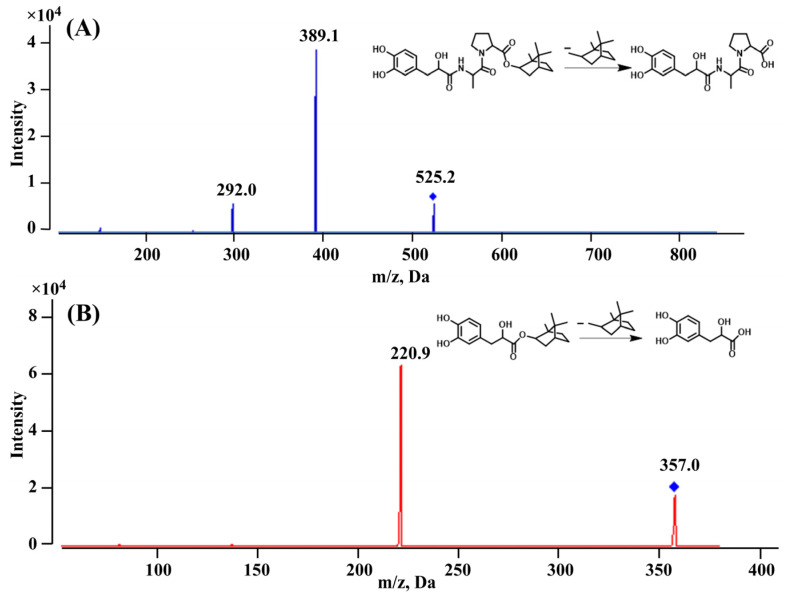
Product ion mass spectra of [M + Na]^+^ for (**A**) DAPB and (**B**) IS.

As illustrated in Figure 1, DBZ was chosen as the internal standard (IS) for its similar retention action to DAPB. Full scans of DAPB and IS were completed in both positive ion mode and negative ion mode, and the results showed that the signal intensity of DAPB is higher in positive ion mode; the ions at *m*/*z* 525.2 and *m*/*z* 357.0 were the peaks for DAPB and IS in the mass spectra, respectively. As shown in Figure 2, after optimization and fragmentation in the collision cell, the ions at *m*/*z* 389.1 and 220.9 were confirmed as the most stable and least disturbed product ions of DAPB and IS, respectively. The compound-dependent parameters, such as the decluttering potential (DP), entrance potential (EP), collision cell exit potential (CXP), and collision energy (CE), were set at 170, 10, 6, and 63 V for DAPB, and 60, 10, 6, and, 20 V for IS.

UPLC parameters including the mobile phase, flow rate, column temperate, and type of column were optimized to achieve an appropriate retention time, high resolution, and improved sensitivity. The optimal mobile phase consisted of deionized water with 0.02% formic acid, and 5 mM NH_4_F (solvent A) and acetonitrile (solvent B). Separation was performed at a flow rate of 0.2 mL/min and with an injection volume of 10 μL with the following gradient elution: 0.0–0.6 min, 45% solvent B; 0.6–2.5 min, 95% solvent B; 2.5–5 min, 45% solvent B.

The pretreatment of plasma samples is crucial to develop an improved selective and sensitive quantitative method. Thus, acetonitrile and methanol were tested for protein deposition; the effect of acetonitrile protein deposition on DAPB was better than that of methanol. Finally, the volume of acetonitrile was optimized, and a volume ratio of 3:1 of acetonitrile to plasma sample was chosen for the precipitation.

### 2.2. Method Validation

#### 2.2.1. Selectivity and Specificity

**Figure 3:**
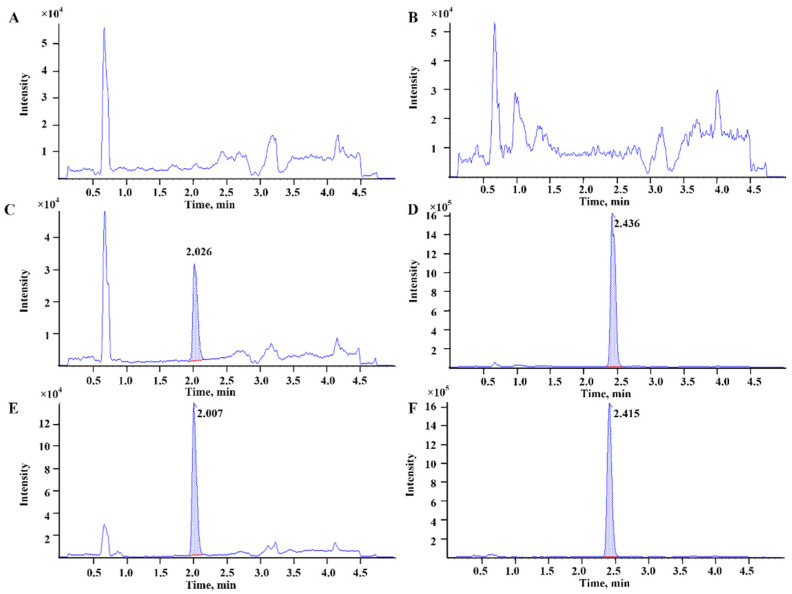
Representative MRM chromatograms of (**A**) DAPB and (**B**) IS in blank rat plasma; (**C**) 2 ng/mL DAPB and (**D**) 200 ng/mL IS in blank rat plasma; (**E**) DAPB and (**F**) IS in rat plasma samples 15 min after oral administration of DAPB at 30 mg/kg.

Selectivity and specificity tests were conducted using blank rat plasma with or without DAPB and rat plasma 1 h after administration with DAPB. As shown in Figure 3, no endogenous substance interfered with the retention times of DAPB and IS in the rat plasma.

#### 2.2.2. Linearity and LLOQ

The calibration curves for DAPB were linear over a concentration range of 2–1000 ng/mL. The regression equation was Y = 0.0149X + 0.1034 (r2 = 0.9966), where Y is the peak area ratio of DAPB to IS, and X is the concentration of DAPB. The LLOD of DAPB was calculated to be 2 ng/mL, which satisfies the requirements of pharmacokinetic studies.

#### 2.2.3. Precision and Accuracy

The intra- and inter-day precision and accuracy tests for DAPB were carried out in variable-concentration quality control samples (2, 6, 500, 800, and 1000 ng/mL). As illustrated in Table 1, the accuracy of the intra- and inter-day samples ranged from −9.5 to 6.8% and from −7.1 to 4.3%, respectively. The precision of the intra- and inter-day samples ranged from 1.3 to 12.7% and from 1.6 to 10.2%, respectively. The results showed that the quantitative method for DAPB is reproducible and reliable.

#### 2.2.4. Extraction Recovery and Matrix Effect

The matrix effect was determined in six replicates by measuring the ratio of DAPB added to plasma to that in acetonitrile. The values of the matrix effect were 103.22%, 95.88%, and 99.43% for DAPB concentrations of 10, 500, and 1000 ng/mL, respectively. These results show (Table 2 and Table 3) that no significant matrix effect was observed under the present conditions.

The extraction recoveries of DAPB were 80.61 ± 5.68, 97.16 ± 2.61, and 96.32 ± 1.76 for DAPB concentrations of 10, 500, and 1000 ng/mL, respectively.

#### 2.2.5. Stability

Esterase widely exists in the blood, serum, and plasma of organisms. Therefore, DAPB may be unstable and easily hydrolyzed by plasma esterases owning to the ester group. In the method-development process, the stability of DAPB in whole blood and plasma was tested in detail. Low temperature is an important factor for maintaining the stability of the analyte; therefore, in our study, an ice-water bath was used to provide cooling conditions. Sodium fluoride (NaF), a typical esterase inhibitor, was pre-added in the collected blood samples to prevent the hydrolysis of DAPB. The stability data are summarized in Table 4. In the plasma with NaF added, DAPB was stable after being placed at room temperature for 4 h, −80 °C for 30 days, and then subject to three freeze/thaw cycles. In addition, the post-preparative plasma sample stored in an autosampler for 24 h remained stable. The dilution capability results for DAPB illustrated that RE% varied from −9.7 to 4.2 with a RSD of <7.0%, suggesting that this quantitative method is sufficient for detection in samples with concentrations above the ULOQ with five-fold dilution.

### 2.3. Pharmacokinetic Study in Rats

**Figure 4:**
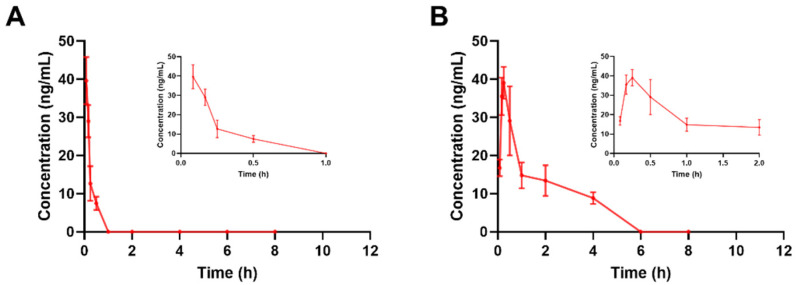
Mean plasma concentration–time profiles of DAPB in rat plasma after intravenous administration at 6 mg/kg (**A**) and oral administration at 30 mg/kg (**B**).

The validated method was applied to a pharmacokinetic study of DAPB in rats. For an intravenous administration of 6 mg/kg, the plasma concentration of DAPB versus time profile is shown in Figure 4 and the pharmacokinetic parameters are summarized in Table 5. DAPB was quickly eliminated from plasma, with a short t_1/2_ value of 0.19 ± 0.01 h. The plasma concentration–time profile for DAPB after oral administration (30 mg/kg) is shown in Figure 4B. The results indicate that DAPB was rapidly absorbed in plasma and could be detected at 5 min after oral administration; it reached a peak at 14.67 ± 4.54, with a Cmax of 33.25 ± 10.87 ng/mL. Our data suggested that the content of DAPB was very low in the rat plasma after intravenous or oral administration, which may be related to the ester bond that can be easily hydrolyzed by esterase in the body. The metabolites of DAPB will be further studied in subsequent research.

## 3. Discussion

To our knowledge, a HPLC–UV method was previously established to quantify DAPB in organic solvents but it has not proved useful for quantifying the content of DAPB in biological samples [24]. The matrices analyzed in methanol and rat plasma are significantly different, and the endogenous substances in plasma may interfere with the determination of DAPB. Herein, a UPLC–MS/MS method was developed and finally validated by evaluating selectivity, specificity, linearity, LLOQ, precision, accuracy, extraction recovery, the matrix effect, and stability. UPLC–MS/MS has become a widely selected practical technology in biological analysis due to its selectivity and sensitivity [25]. In our present research, the chromatographic analysis showed that the running time is very short, at 5 min.

In addition, the procedure of plasma sample pretreatment was simple and easy to carry out. The plasma sample extracted with acetonitrile had excellent recovery within an acceptable range, and no significant interference was observed at the retention time of DAPB and IS. The rat plasma processing method can remove most of the interferents in plasma, such as proteins and other endogenous substances, which plays an important role in achieving specificity and accuracy.

In terms of the analytes, the biggest challenge is to maintain their stability during sampling and determination. Esterase, an enzyme that can hydrolyze ester compounds, is mainly present in plasma and various tissues in the human body [26]. The presence of esterase may affect the stability of the analyte in plasma; therefore, the stability of DAPB in whole blood and plasma was tested in detail in the presence of NaF, a typical esterase inhibitor [27]. The results showed that the tested samples were stable after being stored at room temperature for 2 h, being subjected to repeated three freeze/thaw cycles, and placed in an autosampler rack (4 °C) for 24 h and at 80 °C for 30 days.

The proposed UPLC–MS/MS method was applied to determine the plasma concentration of DAPB in rats following intravenous (5 mg/kg) and oral (30 mg/kg) administration. The sensitivity and specificity of this method were sufficient to accurately characterize the plasma pharmacokinetics of DAPB in rats. DAPB was rapidly eliminated from plasma, which results from the chemical properties of DAPB, the potential hydrolysis in the gastrointestinal tract, and poor permeability through the intestinal epithelial membrane. The metabolites of DAPB will be further studied in subsequent research. In addition to pharmacokinetic studies, this newly developed and validated method can also be applied to other fields, providing insights for further research on DAPB.

## 4. Materials and Methods

### 4.1. Chemicals and Reagents

DAPB (purity > 99.5%) was chemically synthesized and provided by Xi’an Key Laboratory of multi-synergistic antihypertensive innovative drug development of Xi’an Medical University (Xi’an, China). DBZ was kindly provided by the Key Laboratory of Resource Biology and Biotechnology in Western China, Northwest University (Xi’an, China). HPLC-grade acetonitrile, formic acid, and methanol were purchased from Thermo Fisher Scientific Co. (Beijing, China). HPLC-grade NH_4_F was purchased from Merck (Merck, Germany). All other chemicals were of HPLC-grade quality.

### 4.2. UPLC–MS/MS Instrument and Conditions

The UPLC–MS/MS system consisted of a Shimadzu UPLC instrument (two binary pumps LC20ADXR, temperature controller, and autosampler) and an AB SCEIX TRIPLE QUAD 6500 mass. An Agilent SB-C18 column (1.8 μm, 3.0 mm × 50 mm) maintained at 40 °C was applied for chromatographic separation. The mobile phase consisted of deionized water with 0.02% formic acid, and 5 mM NH_4_F (solvent A) and acetonitrile (solvent B). Separation was performed at a flow rate of 0.2 mL/min and with an injection volume of 10 μL with the following gradient elution: 0.0–0.6 min, 45% solvent B; 0.6–2.5 min, 95% solvent B; 2.5–5 min, 45% solvent B.

Mass quantification was carried out using positive ion electrospray ionization (ESI) in multiple reaction monitoring (MRM) mode. The MRM transitions of DAPB and IS were 525.1→389.1 and 357.0→220.9, respectively. The optimal parameters for MS were as follows: curtain gas, 25 psi; temperature, 550 °C; ion source gas 1 (GS1), 50 psi; ion source gas 2 (GS2), 40 psi; ion spray voltage, 5000 V; collision-activated dissociation, 9 psi. The compound-dependent parameters the decluttering potential (DP), entrance potential (EP), collision cell exit potential (CXP), and collision energy (CE) were set at 170, 10, 6, and 63 V for DAPB, and 60, 10, 6, and 20 V for IS. Data acquisition and analysis were conducted using Analyst software (version 1.6.3).

### 4.3. Preparation of Calibration Standards and Quality Control Samples

Standard stock solutions of DAPB and IS were prepared in acetonitrile at the same concentration of 1 mg/mL. Then, the stocking solutions were diluted with acetonitrile to prepare a series of working standard solutions. Similarly, quality control (QC) working solutions were prepared using an independent stocking solution. All the solutions were stored at −20 °C.

Calibration standard samples were made by adding 10 μL of working standard solution, 10 μL of IS working solution (1 μg/mL), and 280 μL of acetonitrile to 100 μL of blank plasma pretreated with 1 μL of NaF (5 mg/mL). The final concentrations of DAPB in plasma were 2.00, 5.00, 10.00, 20.00, 50.00, 100.00, 200.00, 500.00, and 1000.00 ng/mL. QC samples (2.00, 10.00, 500.00, 800.00, and 1000.00 ng/mL for DAPB) were prepared in the same manner as the calibration standard samples. All calibration standard samples and QC samples were centrifuged twice at 12,000 rpm for 5 min at 4 °C after vortexing for 30 s. For analysis, 10 μL of supernatant was injected into the UPLC–MS/MS apparatus.

### 4.4. Method Validation

According to the US Food and Drug Administration (FDA) guidelines (2019), the optimized bioanalytical method for DAPB was fully validated by evaluating selectivity, specificity, linearity, LLOQ, precision, accuracy, extraction recovery, the matrix effect, and stability.

#### 4.4.1. Selectivity and Specificity

To investigate the potential interference with endogenous components in plasma, the tests of selectivity and specificity were performed on the plasma collected from six rats with or without DAPB and IS. The chromatographic peaks of DAPB and IS were assessed according to their retention times and MRM responses.

#### 4.4.2. Linearity and LLOQ

The linearity of DAPB in plasma was investigated using a nine-point calibration curve by plotting the ratio of the peak area of the analyte to that of IS versus the concentrations of the calibration standards. A weighted (1/x^2^) linear least-squares regression model was chosen to calculate the slope and correlation coefficient (R^2^) from the calibration curves. LLOQ was defined as the lowest concentration of analyte that can be accurately measured on the calibration curve.

#### 4.4.3. Precision and Accuracy

To evaluate precision and accuracy, five different concentrations of QC samples (2, 10, 500, 800, and 1000 ng/mL) in replicates of six were detected on three consecutive days (inter-day) and on the same day (intra-day). Accuracy was expressed as the relative error (RE) by calculating the ratio of the measured concentration to the spiked concentration, and precision was expressed by calculating the relative standard deviation for the QC samples of the inter- and intra-day replicates. The precision and accuracy of all the QC samples should be within ±15%, except for LLOQ, which should not exceed 20%.

#### 4.4.4. Extraction Recovery and Matrix Effect

The extraction recovery was calculated by comparing the mean peak areas of the QC samples at different concentration levels (10, 500, and 1000 ng/mL) in six replicates with post-extracted blank plasma spiked with DAPB at three QC concentrations (*n* = 6).

The matrix effect was measured by calculating the ratio of the peak area in the presence of plasma (measured after extraction from blank plasma and with the addition of analyte and IS) to the corresponding peak area in the working solution at three concentration levels (10, 500, and 1000 ng/mL). The matrix factor calculated from six batches of rat plasma should not exceed 15%.

#### 4.4.5. Stability

The stability of DAPB in rat plasma was assessed by using QC samples at three concentrations in six replicates following three freeze/thaw cycles, short-term storage (room temperature for 2 h), long-term storage (−80 °C for 30 consecutive days), and autosampler rack storage (4 °C for 24 h). In freeze/thaw cycle tests, the QC samples were frozen and stored at −80 °C and then thawed at room temperature; this was repeated three times. The values of stability were calculated by comparing the concentrations of the QC samples following each storage period with the concentrations of the freshly prepared QC samples in the same analytical run.

A dilution test was performed to verify that sample dilution did not affect the accuracy and precision of the optimized method for qualifying DAPB. Two concentration levels of QC samples (500 and 1000 ng/mL) were diluted five-fold with blank rat plasma. Dilution capability was assessed by comparing the measured concentrations of the diluted samples and multiplying the diluted factor by the nominal value.

### 4.5. Pharmacokinetic Study

Sprague–Dawley (SD) rats (180–200 g) were purchased from the Experimental Animal Center of Xi’an Jiaotong University (permit number SCXK 2017–003) and housed under controlled conditions (25 °C, 50 ± 5% humidity, and 12 h dark–light cycles). The animal protocol was approved by the Animal Ethics Committee of Xi’an Medical College. After acclimation for one week, 10 rats were fasted overnight and randomized into two groups. For the intravenous administration group, DAPB prepared in saline containing 5% DMSO was injected in the tail vein at a dose of 6 mg/kg. For the oral administration group, rats were given DAPB dissolved in 10% (2-hydroxypropyl)-β-cyclodextrin in water (10% HP-β-CD solution) containing 20% PEG200 and 20% propylene glycol at a dose of 30 mg/kg orally. Blood samples were collected in heparinized tubes pretreated with NaF via the ophthalmic venous plexus before and at 0.083, 0.17, 0.25, 0.50, 1.00, 2.00, 4.00, and 8.00 h after dosing. After centrifugation, the supernatant plasma was obtained and stored at −80 °C for further treatment. For the preparation of the plasma samples, 10 μL of the IS working solution (1 μg/mL), 1 μL of NaF (5 mg/mL), and 290 μL of acetonitrile were added to 100 μL of the plasma samples. Subsequently, the mixed plasma samples were centrifuged twice at 16,000× *g* for 5 min at 4 °C after vortexing for 30 s. For analysis, 10 μL of the supernatant was injected into the UPLC–MS/MS apparatus.

## 5. Conclusions

A simple, specific, and sensitive UPLC–MS/MS method for determining DAPB in the plasma of rats was developed and validated. The validated method was successfully applied to the pharmacokinetic study of DAPB in rats after oral administration and intravenous administration.

## Figures and Tables

**Table 1:** Accuracy and precision of DAPB in rat plasma (*n* = 6).

Normial Conc. (ng/mL)	Intra-Day			Inter-Day		
	Measured Conc. (Mean ± SD, ng/mL)	Accuracy (RE, %)	Precision (RSD, %)	Measured Conc. (Mean ± SD, ng/mL)	Accuracy (RE, %)	Precision (RSD, %)
2	1.81 ± 0.23	−9.5	12.7	1.86 ± 0.19	−7.1	10.2
10	10.24 ± 0.58	2.4	5.6	9.93 ± 0.45	−0.7	4.5
500	521.88 ± 9.34	4.4	1.8	518.25 ± 8.47	3.7	1.6
800	791.34 ± 10.19	−1.1	1.3	822.67 ± 11.13	2.8	1.4
1000	1068.47 ± 18.92	6.8	1.8	1042.59 ± 21.46	4.3	2.1

**Table 2:** Matrix effect of DAPB in rat plasma (*n* = 6).

Spiked Conc. (ng/mL)	Matrix Effect (Mean ± SD, %)	RSD (%)
10	103.22 ± 8.91	8.6
500	95.88 ± 5.12	5.3
1000	99.43± 7.45	7.5

**Table 3:** Extraction recovery of DAPB in rat plasma (*n* = 6).

Spiked Conc. (ng/mL)	Extraction Recovery (Mean ± SD, %)	RSD (%)
5	80.61 ± 5.68	7.1
500	97.16 ± 2.61	2.7
1000	96.32 ± 1.76	1.8

**Table 4:** Stability of DAPB in rat plasma (*n* = 6).

Stability Conditions	Spiked Conc. (5 ng/mL)		Spiked Conc. (500 ng/mL)		Spiked Conc. (1000 ng/mL)	
	RE (%)	RSD (%)	RE (%)	RSD (%)	RE (%)	RSD (%)
Room temperature for 2 h	−10.2	7.8	−7.5	6.4	1.2	4.9
Three freeze/thaw cycles	−6.4	9.1	2.6	5.7	−2.0	4.5
−80 °C for 30 days	5.3	6.7	−6.2	3.5	−4.6	2.7
Autosampler rack (4 °C) for 24 h	−11.6	9.4	−7.4	5.5	−3.4	7.8
Dilution capability (factor: 5)	-	-	4.2	6.6	−9.7	6.8

**Table 5:** PK parameters of DAPB in rat plasma after intravenous administration at 6 mg/kg and oral administration at 30 mg/kg. Data are expressed as mean ± SD (*n* = 6).

PK Parameters	Unit	Intravenous (Mean ± SD)	Oral (Mean ± SD)
t_1/2_	h	0.19 ± 0.01	1.63 ± 0.36
AUC_0–t_	h·ng/mL	11.77 ± 1.66	49.62 ± 7.92
AUC_0–∞_	h·ng/mL	13.78 ± 2.10	53.87 ± 8.34
Vss	L/kg	910.03 ± 24.38	264.73 ± 65.86
CL	mL/h/kg	440,589.57 ± 67,015.75	89,992.36 ± 10,292.61
T_max_	min	-	14.67 ± 4.54
C_max_	ng/mL	-	33.25 ± 10.87
F	%	-	78.20

## Data Availability

The authors confirm that the data supporting the findings of this study are available within the article.
